# Repeatability of scapular motion tracking in individuals with high body mass index

**DOI:** 10.3389/fspor.2025.1722571

**Published:** 2026-01-12

**Authors:** Angelica E. Lang, Sophia Abiara

**Affiliations:** 1 Canadian Centre for Rural and Agricultural Health, College of Medicine, University of Saskatchewan, Saskatoon, SK, Canada; 2 Department of Medicine, College of Medicine, University of Saskatchewan, Saskatoon, SK, Canada

**Keywords:** double calibration, dynamic, functional, kinematics, obesity, shoulder

## Abstract

**Introduction:**

Scapular motion tracking is essential for understanding shoulder kinematics, but its utility in individuals with high body mass index (BMI) requires further investigation. The purpose of this study was to define repeatability of skin-based scapular motion tracking in individuals with BMI ≥ 30 using two calibration procedures.

**Methods:**

Nine participants (mean BMI (SD): 42.1 (7.9)) completed two sessions involving planar arm elevations and functional arm-focused tasks. Scapular motion was tracked with the acromial marker cluster, and scapular kinematics were calculated from single and double calibration procedures. Repeatability of discrete scapular angles at 15° increments of humeral elevation was evaluated using intraclass correlation coefficients (ICCs), and minimal detectable change (MDC). Differences in angle waveforms were also explored with statistical parametric mapping.

**Results:**

Findings suggest that scapular motion tracking is feasible and repeatable for upward rotation and internal rotation in individuals with high BMI (ICCs = 0.50-0.90; MDCs = 5 to 15°). The single calibration method underestimated upward and internal rotation as compared to the double calibration method, particularly at higher humeral elevations.

**Discussion:**

Although BMI was used as a proxy for body composition, this study supports scapular motion tracking with the acromial marker cluster in populations where soft tissue artifact may be more pronounced.

## Introduction

Scapular motion tracking is essential to understand fundamental shoulder movement. However, there are many inherent challenges for scapular motion tracking due to the orientation of the scapula on the rib cage. Previous research has meticulously investigated accuracy, reliability, and repeatability of skin-based scapular motion tracking methods and generally supports the use of these methods (1–10). While the range of error or repeatability can be large, standard error of measurement (SEM) and minimal detectable change (MDC) are often reported to be approximately 2°–7° and 6°–20°, respectively, in scapular motion tracking validation studies (4, 11, 12). However, the vast majority of these studies have been completed with participants who were young, healthy, and relatively fit and homogeneous. The application of scapular motion tracking to people with differing characteristics would further confirm the validity of these methods.

Body composition is one such individual characteristic that may have an important effect on kinematic outcomes. Main sources of measurement error for marker-based motion capture are due to segment tracking limitations, such as soft tissue artifact or inaccurate anatomical landmark identification (13, 14). These error sources can be further amplified in the presence of more body tissue surrounding the bones or segments of interest, including adipose tissue or developed muscle (15). More soft tissue is expected to contribute to greater skin movement artifact when tracking the bone movement, which could affect biomechanical outcomes (16). Landmark location through palpation, the typical approach for defining the location of relevant anatomical markers for motion capture, may also be more difficult in the presence of more body tissue. Still, motion capture of the lower limb has been demonstrated to be acceptable, defined as SEM <5°, in an obese population (17). Additionally, certain developments in scapular motion tracking, specifically the double calibration method (18), may account for errors related to soft tissue artifact, indicating scapular motion tracking could be acceptable in obese individuals. Therefore, the purpose of this study was to assess the repeatability of scapular motion tracking and compare scapular kinematic outcomes from single and double calibration methods from a sample with a high body mass index (BMI).

## Methods

### Participants

Participants were recruited from a convenience sample from the University of Saskatchewan community. Participants self-selected based on advertisements on University digital and physical notice boards. The target population for this study was individuals between the ages of 18 and 35 (8, 19) with a BMI of 30 or higher, which is defined as “obese” (20). BMI was used as the identifier for this population because it is an easy-to-calculate comparative descriptor that has commonly been used to describe body size. Though BMI does not provide any indication of the body composition, this measure was chosen because large body size can be implicated in skin movement artifact and difficulty with palpation. Other exclusion criteria included any upper limb pain or discomfort in the past six months, previous shoulder surgery, or presence of known musculoskeletal disorders, such as arthritis. All study procedures were approved by the institutional research ethics board (Bio #2941), and all participants provided informed written consent.

### Procedures

A test-retest study design was used, in which each participant attended two sessions at least one day apart. At the beginning of the first session, height and weight were measured, and arm dominance was recorded.

**Figure 1:**
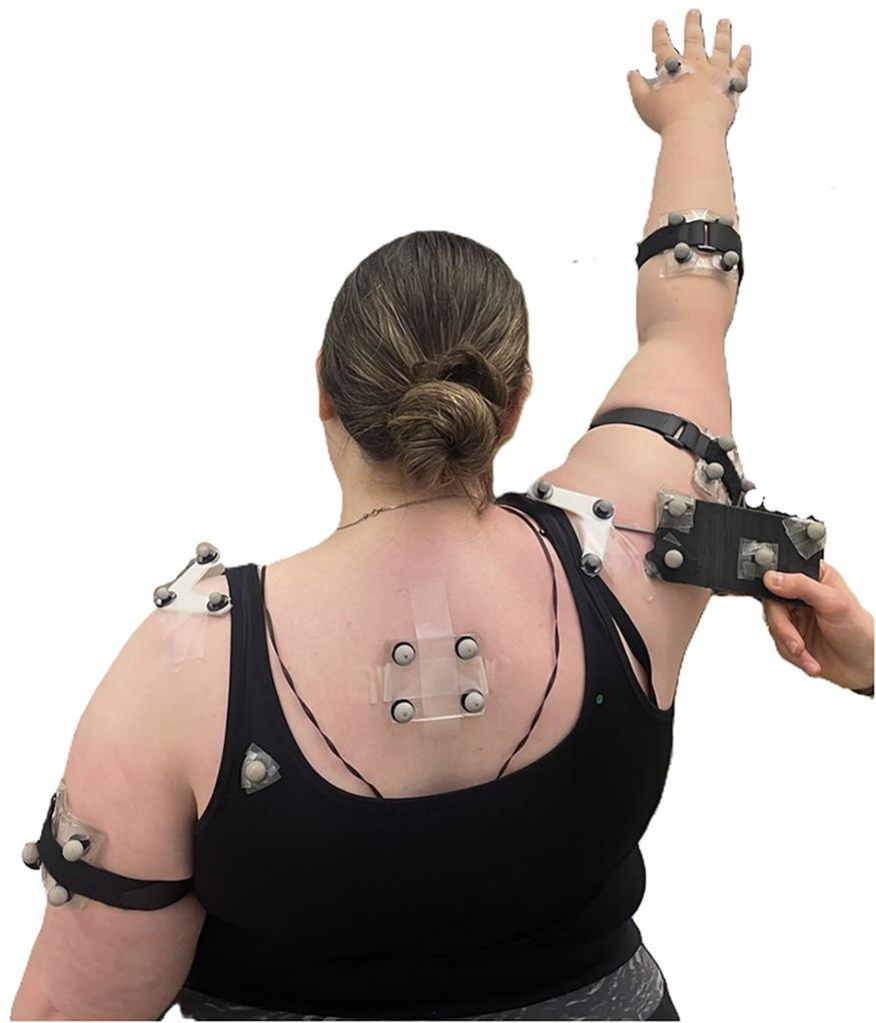
Scapular calibration during the maximum elevation calibration pose.

Reflective markers (individual markers and rigid clusters) were affixed to the torso and dominant arms of each participant, as per International Society of Biomechanics (ISB) recommendations for individual markers (21) and standard laboratory operating procedures for clusters. Positions of the anatomical markers for the humerus and torso were recorded in a T pose. An acromion marker cluster (AMC) was placed on the most lateral aspect of flat part of the acromion (2, 22). The anatomical landmarks of the scapula (acromial angle, inferior angle, and root of the scapular spine (21) were defined with a digitizer with the arm by their side, and again at maximum arm elevation in the scapular plane (8, 18) (Figure 1). The digitizer is a rigid rectangular body with four reflective markers and a pointed tip at one end. Once the anatomical landmarks were palpated, the digitizer tip was placed at each landmark, and the position was recorded; the point at the tip of the digitizer was defined as the anatomical landmark, relative to the AMC, derived from the orientation and position of the digitizer. Palpations at maximum arm elevation were guided by a scapular locator adjusted to each individual's scapular dimensions (8, 11, 23). The scapular locator was originally introduced to identify scapular positions in static arm postures (23) and has more recently been used for scapular calibrations with inertial measurement units (9, 24). In this study, the scapular locator was not used to record the anatomical points; instead, this rigid body, made of two arms and three prongs, was leveraged to guide anatomical landmark location at the maximum arm elevation position. The prongs were adjusted to each person's scapular landmarks at the neutral arm position, and then the dimensions and relative positions of the three prongs were used to guide palpations of the landmarks in maximum arm elevation. The glenohumeral joint centre, required for the definition of the humerus long axis, was estimated as 60 mm below the acromial marker, the most lateral marker on the AMC (25).

Participants performed several arm-focused movements, including tasks from the Work-Related Activities and Functional Task protocol. Protocol details can be found elsewhere (8, 19, 26). All motion was tracked with a 10-camera Vicon motion capture system (Vicon Motion Systems, Oxford, UK), sampling at 100 Hz. First, each person performed standing planar elevation in the frontal and sagittal planes. Next, three activities of daily living were completed: Comb Hair, Wash Axilla, and Tie Apron. Finally, three work-related movements were performed: Overhead Reach, Forward Transfer, and Overhead Lift. All tasks were completed unilaterally and seated, except for the Tie Apron and Overhead Lift, which were bilateral standing tasks. Each movement was performed three times (three times on each arm for unilateral tasks). All participants received the same standard instructions and brief demonstrations, including instructions to move at a comfortable pace, but motion was otherwise unconstrained to capture natural movement and task performance.

### Analysis

Scapular and humeral kinematics were calculated for the dominant arms with custom Matlab® scripts (24.1.2537033, R2024a). Repetitions were averaged within participants for each task. Scapular angles (internal rotation, upward rotation, and tilt) were extracted from the scapular and torso anatomical coordinate systems, using the joint coordinate system method and a YX'Z' rotation sequence (21). Angles were transformed so that positive values indicate internal rotation, upward rotation, and posterior tilt. Scapular angles were calculated twice with this method: with the single (neutral) calibration and with the double (neutral and maximum) calibration. The single calibration method used the neutral calibration position only to define the relationship of the anatomical landmarks to the AMC. The double calibration method defines the position of the anatomical landmarks relative to the AMC in two positions, in this case neutral and maximum arm elevation, and then linearly interpolates the local relationship between those two positions to find the relative relationship of the landmarks to the AMC at each frame in the dynamic motions (18). Humeral elevation angle was the independent variable for this interpolation and was calculated as the cosine of the angle between the long axis of the humerus and the torso. This angle was used to define movement cycles for the waveform analysis. Generally, a movement cycle began when the humerus moved 5° and ended at peak humeral elevation (19). For the repeatability assessment, scapular orientations at 15° increments of humeral elevation were extracted as relevant for each task, as the range of humeral movement varied with task.

Repeatability was assessed with mean difference between the sessions, intraclass correlation coefficients (ICCs), and MDC (8, 17, 19, 27, 28). Two-way random effects ICC with absolute agreement (ICC_2,1_) values were interpreted based on the Shrout and Fleiss (29) framework: <0.40 = poor; 0.40–0.59 = fair, 0.60–0.74 = good, >0.74 = excellent. A threshold for MDC acceptability was defined as 14°, equivalent to 5° SEM, as per previous BMI-related reliability research (17). Repeatability outcomes were descriptively compared between calibration procedures.

Angle waveforms were compared with two-way repeated measures ANOVA statistical parametric mapping (SPM) (*p* < 0.05) to assess differences between calibration procedures and sessions across the full waveform (8, 30, 31), with normalized movement cycle (0%–100%) on the *x*-axis.

## Results

Nine participants (4 female) completed both sessions for this study. The mean BMI was 42.1 kg/m^2^ (range: 35.1–53.4; standard deviation: 7.9), and the mean age was 24.2 years (range: 21–31; standard deviation: 3.6). Two participants were left hand dominant.

### Repeatability

Repeatability outcomes were generally good for both single and double calibration procedures (Tables 1–3). For upward and internal rotation, MDCs were under the 14° threshold for most combinations of task, elevation level, and calibration procedure. ICCs were also good to excellent for both degrees of freedom. For upward rotation, the single calibration had slightly improved ICCs (mean ICC: 0.67 double vs. 0.85 single), while for internal rotation, double calibration had slightly improved ICCs (mean ICC: 0.73 double vs. 0.61 single). For tilt angles, MDC values were generally higher (approximately 12°–19°), and ICCs ranged from poor to excellent for both calibration methods (mean ICC: 0.52 double vs. 0.48 single). Notably, MDCs were highest for all degrees of freedom at 120° of humeral elevation during flexion (sagittal plane elevation) with the single calibration method, although the results for double calibration were also high at this point, particularly for tilt.

**Table 1:** Difference and repeatability between sessions for upward rotation at each humeral elevation level.

Upward rotation (°)		45			60			75			90			120		
		Mean	ICC	MDC	Mean	ICC	MDC	Mean	ICC	MDC	Mean	ICC	MDC	Mean	ICC	MDC
Comb Hair	DBL	−0.3	0.7	10.9	0.3	0.7	7.1	0.1	0.6	7.1	0.9	0.7	6.7	–	–	–
	SGL	−2.0	0.9	9.2	−1.9	0.9	7.8	−2.5	0.9	8.6	−2.8	0.9	7.6	–	–	–
Wash Axilla	DBL	0.8	0.7	14.4	–	–	–	–	–	–	–	–	–	–	–	–
	SGL	−0.9	0.8	14.5	–	–	–	–	–	–	–	–	–	–	–	–
Tie Apron	DBL	2.1	0.7	5.2	4.7	0.5	10.6	–	–	–	–	–	–	–	–	–
	SGL	0.4	0.9	4.5	2.2	0.8	6.9	–	–	–	–	–	–	–	–	–
Overhead Reach	DBL	–	–	–	0.4	0.7	9.2	0.5	0.5	9.6	0.9	0.5	9.6	–	–	–
	SGL	–	–	–	−2.7	0.9	9.3	−2.3	0.8	11.2	−2.6	0.8	11.1	–	–	–
Forward Transfer	DBL	−1.8	0.7	12.9	1.6	0.7	8.8	–	–	–	–	–	–	–	–	–
	SGL	−3.5	0.8	13.0	−0.4	0.9	10.0	–	–	–	–	–	–	–	–	–
Overhead Lift	DBL	–	–	–	1.6	0.5	12.3	2.6	0.7	9.3	0.9	0.6	11.0	–	–	–
	SGL	–	–	–	−0.6	0.5	14.4	−0.2	0.5	12.5	−3.2	0.7	12.6	–	–	–
Abduction	DBL	0.5	0.8	5.1	1.1	0.8	5.4	1.5	0.8	5.6	1.5	0.9	5.3	1.3	.97	3.4
	SGL	−0.9	0.9	5.2	−0.9	0.9	5.1	−1.3	0.9	5.2	−2.1	0.9	8.2	−0.4	.85	12.7
Flexion	DBL	0.1	0.8	6.0	0.5	0.8	6.3	1.4	0.6	7.9	2.6	0.5	10.9	5.6	.71	10.8
	SGL	−1.2	0.9	7.2	−1.5	0.9	6.7	−1.3	0.9	6.4	−1.2	0.9	6.5	−1.2	.85	14.5

DBL, double calibration; SGL, single calibration; Mean, mean difference; ICC, intraclass correlation; MDC, minimal detectable change.

**Table 2:** Difference and repeatability between sessions for internal rotation at each humeral elevation level.

Internal rotation (°)		45			60			75			90			120		
		Mean	ICC	MDC	Mean	ICC	MDC	Mean	ICC	MDC	Mean	ICC	MDC	Mean	ICC	MDC
Comb Hair	DBL	−2.2	0.8	9.0	−0.6	0.8	9.6	−1.1	0.9	10.5	−2.1	0.9	10.9	–	–	–
	SGL	−0.6	0.8	8.2	1.7	0.7	10.1	1.9	0.7	10.5	2.3	0.8	10.8	–	–	–
Wash Axilla	DBL	−1.6	0.6	14.8	–	–	–	–	–	–	–	–	–	–	–	–
	SGL	−0.7	0.5	14.4	–	–	–	–	–	–	–	–	–	–	–	–
Tie Apron	DBL	−2.9	0.8	11.6	−4.6	0.8	15.1	–	–	–	–	–	–	–	–	–
	SGL	−1.4	0.8	11.2	−2.9	0.7	13.8	–	–	–	–	–	–	–	–	–
Overhead Reach	DBL	–	–	–	−2.8	0.8	11.9	−0.1	0.7	14.3	−2.0	0.8	13.2	–	–	–
	SGL	–	–	–	0.0	0.8	12.9	3.0	0.7	13.8	2.3	0.8	11.6	–	–	–
Forward Transfer	DBL	0.9	0.5	12.2	−1.3	0.6	11.7	–	–	–	–	–	–	–	–	–
	SGL	2.7	0.2	12.4	0.7	0.1	12.7	–	–	–	–	–	–	–	–	–
Overhead Lift	DBL	–	–	–	−0.6	0.9	9.1	−1.5	0.9	9.8	−1.6	0.9	11.5	–	–	–
	SGL	–	–	–	1.6	0.7	10.8	1.9	0.8	8.7	2.6	0.8	9.2	–	–	–
Abduction	DBL	−1.4	0.7	10.3	−2.7	0.8	10.9	−3.5	0.8	11.7	−4.7	0.9	13.4	−0.2	.92	12.3
	SGL	0.1	0.6	10.9	−0.6	0.7	10.7	−0.5	0.8	10.0	−0.4	0.8	9.8	1.5	.48	15.5
Flexion	DBL	−1.9	0.7	10.9	−2.5	0.7	12.9	−3.3	0.7	14.6	−3.8	0.8	15.3	−1.1	.89	15.2
	SGL	−0.3	0.7	11.7	−0.2	0.6	14.1	−0.2	0.6	16.3	0.2	0.6	18.3	2.7	.58	26.0

DBL, double calibration; SGL, single calibration; Mean, mean difference; ICC, intraclass correlation; MDC, minimal detectable change.

**Table 3:** Difference and repeatability between sessions for tilt at each humeral elevation level. .

Tilt (°)		45			60			75			90			120		
		Mean	ICC	MDC	Mean	ICC	MDC	Mean	ICC	MDC	Mean	ICC	MDC	Mean	ICC	MDC
Comb Hair	DBL	3.0	0.3	18.9	3.1	0.3	19.2	3.1	0.4	18.7	3.8	0.6	17.1	–	–	–
	SGL	5.0	0.5	17.9	6.2	0.4	18.3	6.6	0.3	18.4	7.1	0.3	17.1	–	–	–
Wash Axilla	DBL	2.7	0.4	17.4	–	–	–	–	–	–	–	–	–	–	–	–
	SGL	4.6	0.5	17.0	–	–	–	–	–	–	–	–	–	–	–	–
Tie Apron	DBL	4.3	0.6	10.4	4.1	0.7	10.4	–	–	–	–	–	–	–	–	–
	SGL	15.2	0.3	14.6	16.8	0.4	15.5	–	–	–	–	–	–	–	–	–
Overhead Reach	DBL	–	–	–	0.6	0.1	15.6	3.1	0.2	16.7	4.2	0.5	16.1	–	–	–
	SGL	–	–	–	5.2	0.2	21.4	6.5	0.3	16.4	7.3	0.3	16.6	–	–	–
Forward Transfer	DBL	3.8	0.3	15.4	2.0	0.4	15.9	–	–	–	–	–	–	–	–	–
	SGL	4.9	0.5	14.5	5.3	0.6	14.4	–	–	–	–	–	–	–	–	–
Overhead Lift	DBL	–	–	–	4.1	0.4	18.9	5.2	0.6	16.0	3.0	0.9	13.2	–	–	–
	SGL	–	–	–	15.8	0.3	18.7	17.3	0.4	17.6	17.2	0.4	18.9	–	–	–
Abduction	DBL	2.5	0.7	12.9	2.5	0.7	12.9	2.5	0.8	12.2	3.2	0.8	14.2	2.5	.77	18.1
	SGL	5.0	0.7	14.3	5.5	0.6	14.2	5.8	0.6	13.2	6.4	0.4	15.4	2.5	.77	15.3
Flexion	DBL	2.4	0.5	16.5	2.4	0.5	19.2	2.4	0.5	20.2	2.3	0.6	20.8	−0.4	.66	23.1
	SGL	4.7	0.7	14.4	5.4	0.6	16.3	6.0	0.6	17.8	6.2	0.5	19.0	4.0	.90	38.3

DBL, double calibration; SGL, single calibration; Mean, mean difference; ICC, intraclass correlation; MDC, minimal detectable change.

### Waveforms

**Figure 2:**
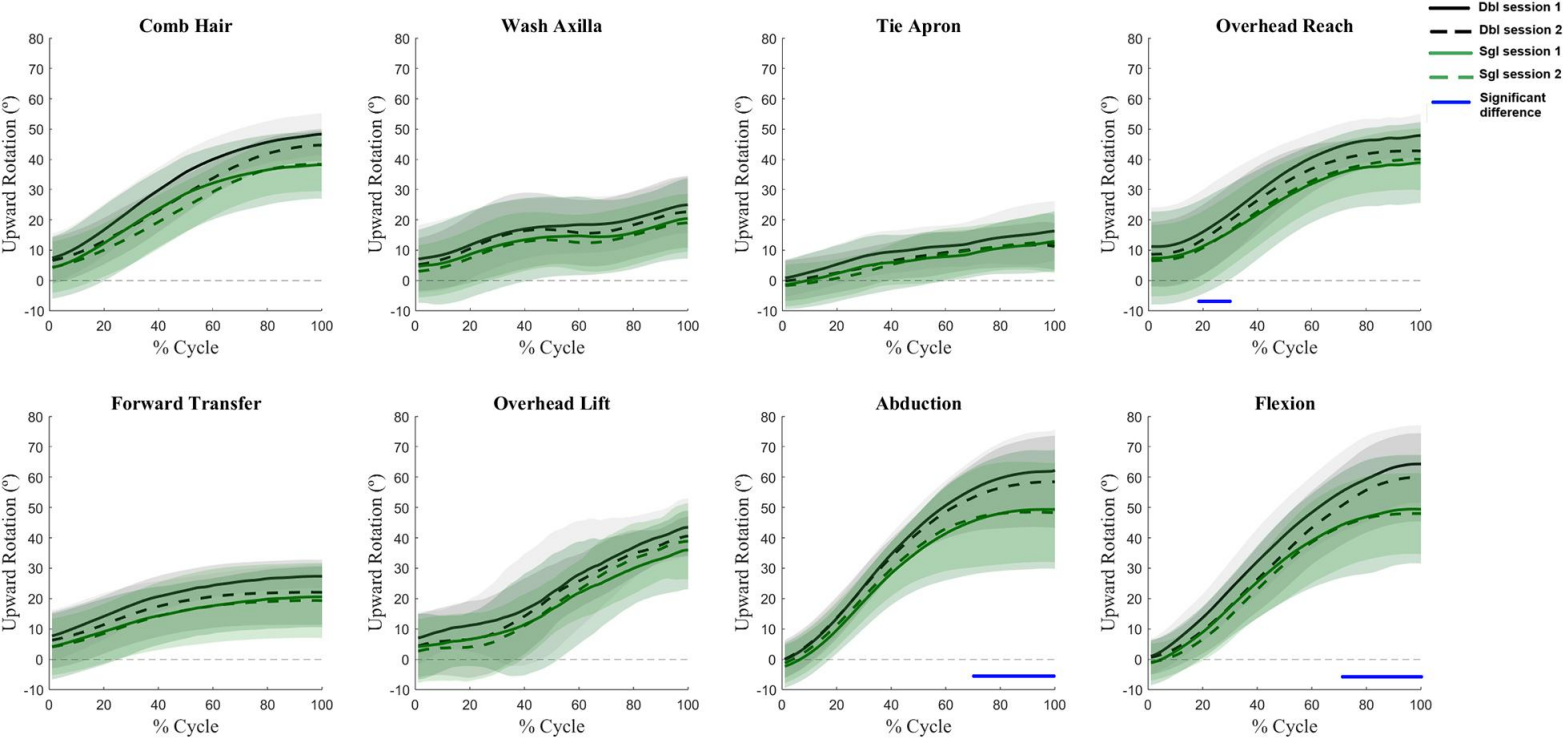
Waveform comparisons of upward rotation between sessions and calibration procedures. In each subplot, the mean waveforms for the double calibration method are represented by black lines and single calibration method by green lines. The shaded areas represent one standard deviation. The solid blue lines at the bottom of subplots indicate the portion of the waveforms in which main effects of calibration procedures were present (i.e., the green lines were significantly different from the black lines).

**Figure 3:**
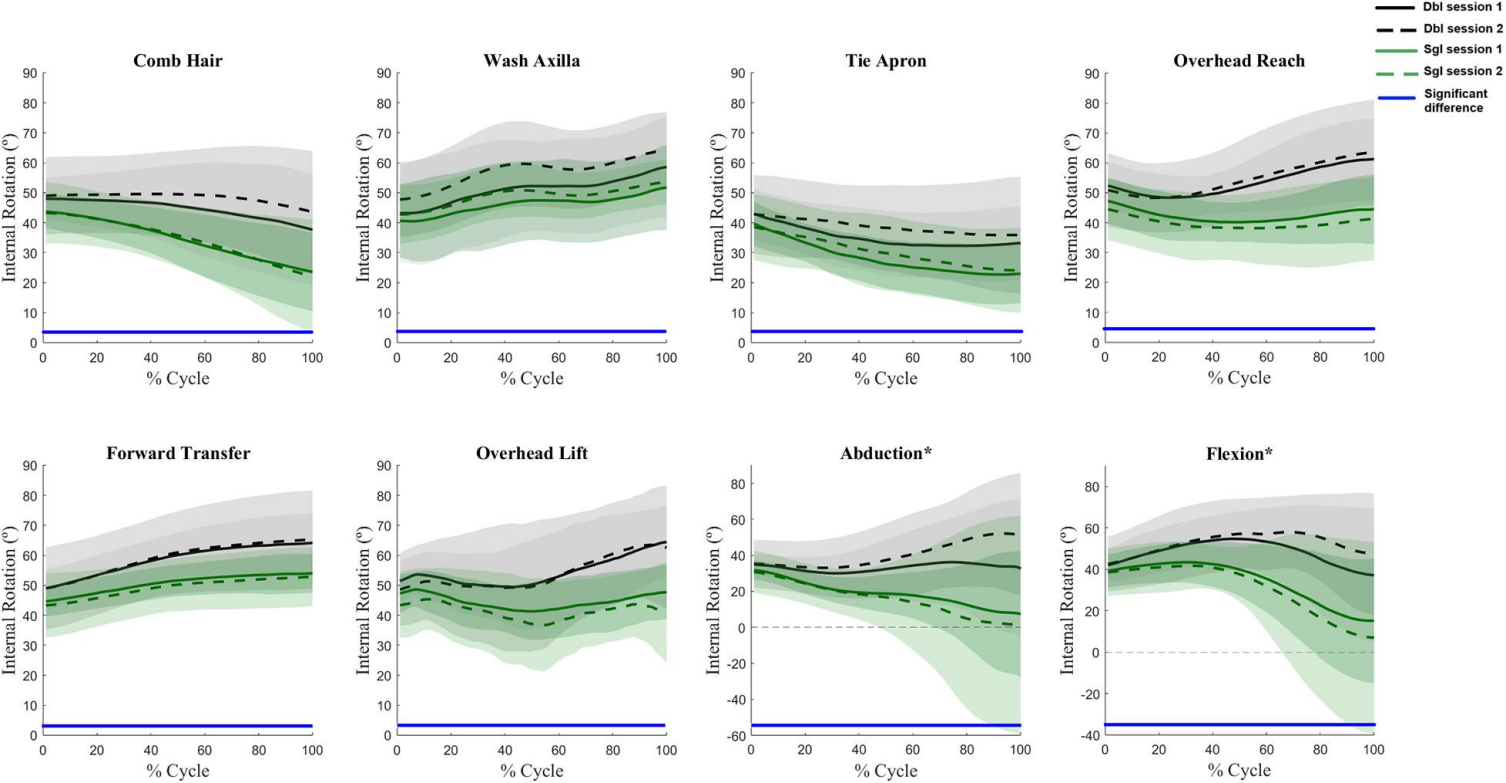
Waveform comparisons of internal rotation between sessions and calibration procedures. In each subplot, the mean waveforms for the double calibration method are represented by black lines and single calibration method by green lines. The shaded areas represent one standard deviation. The solid blue lines at the bottom of subplots indicate the portion of the waveforms in which main effects of calibration procedures were present (i.e., the green lines were significantly different from the black lines). *indicate subplots that have a different *y* axis magnitude to accommodate the standard deviation spread.

**Figure 4:**
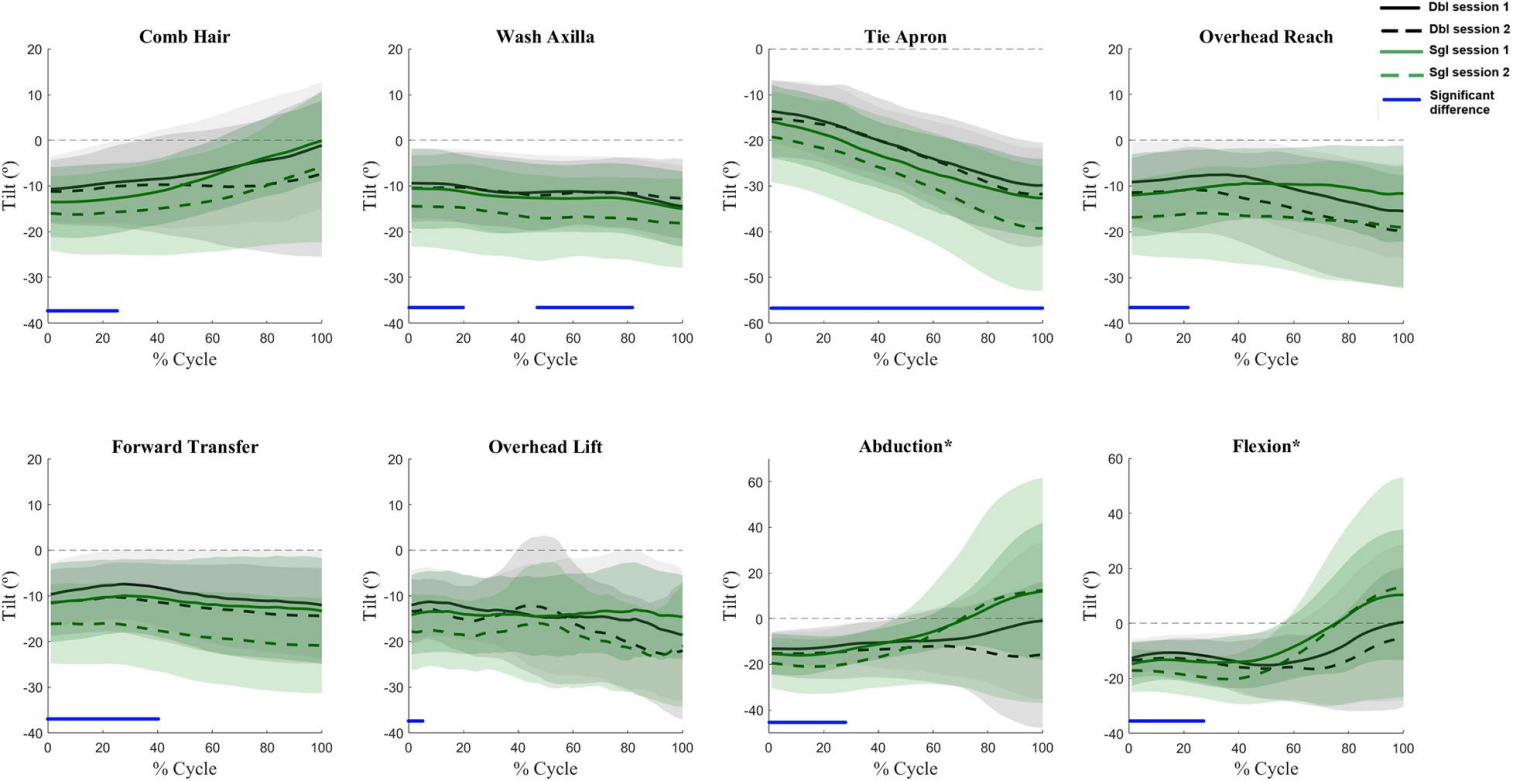
Waveform comparisons of internal rotation between sessions and calibration procedures. In each subplot, the meanwaveforms for the double calibration method are represented by black lines and single calibration method by green lines. The shaded areas represent one standard deviation. The solid blue lines at the bottom of subplots indicate the portion of the waveforms in which main effects of calibration procedures were present (i.e., the green lines were significantly different from the black lines). *indicate subplots that have a different *y* axis magnitude to accommodate the standard deviation spread.

Angle waveforms were not affected by the interaction of session and calibration procedure, nor the main effect of session. Main effects of calibration procedures were present for several tasks for all three scapular angles. Generally, the single calibration method underestimated upward rotation as compared to the double calibration method (Figure 2) with differences most pronounced at the high humeral elevations. Single calibration also resulted in lower internal rotation values (Figure 3) as compared to double calibration throughout all tasks. For tilt, significant differences occurred at lower levels of elevation in all tasks; however, variation also increased substantially at higher levels of arm elevation in the planar elevations (Figure 4).

## Discussion

Despite the wide-ranging applications of motion tracking, the repeatability in people with differing BMIs, and presumably differing body compositions, has been rarely examined. Overall, the findings from this study indicate that the scapulae of people with high BMIs can be tracked as effectively as other populations during a range of arm movements.

Repeatability and reliability of scapular kinematic measurement are important considerations for shoulder motion capture. Previous research has similarly assessed the reliability of scapular kinematic measurement (1, 4, 8, 12, 27, 28, 32, 33); however, the highest average BMI of the sample in these studies was 28 (27), considerably lower than the current study average (42). Additionally, the tested movements are often more highly constrained, largely focused on controlled planar arm elevation, although functional movements and activities of daily living have been tested in some research (8, 12). As noted, SEMs and MDCs can range from 2° to 7° and 6° to 20°, respectively (4, 11, 12). With the exception of tilt at the high levels of elevation during flexion and abduction, the current study values are within this range that was previously reported in these more constrained tasks, indicating that scapular motion tracking may be as effective in a sample with high BMI in less constrained, multiplanar movements.

Repeatability results varied with scapular angle. Upward rotation angles were the most consistent between sessions, followed by internal rotation, and then tilt. Upward rotation has consistently been identified as the most reliable angle with skin-based motion tracking (4, 8, 12). High error levels for internal rotation and tilt may be due to their higher susceptibility to skin movement, regardless of body size, or the more pronounced effect of posture on these angles. Scapular resting position on the thorax has a pronounced influence on internal rotation and tilt (34), and postural differences between days could contribute to the higher variability. In the current study, the MDCs for tilt were consistently high and often above the 14° threshold, suggesting that tilt outcomes from individuals with high BMI should be considered with caution.

Both double and single calibration procedures resulted in similar repeatability outcomes, but there were differences in angle magnitudes between the procedures for many tasks. Previous research indicates that the double calibration procedures are more accurate than single calibrations, particularly at high levels of arm elevation (5, 6, 18, 35). The current findings indicate largest differences occur for upward rotation and tilt as the arm elevates to high levels. However, differences in internal rotation were present throughout full movement cycles for all tasks, with the single calibration values lower than the double calibration values. Internal rotation errors are typically higher with a single calibration (5, 35), suggesting that this degree of freedom may be more susceptible to skin movement artifact in this population, which is better accounted for with the double calibration method.

Although body composition was not explicitly measured, the sample characteristics indicate that this is a unique dataset. All participants in this study had a BMI of 35 or above, corresponding to Class 2 or Class 3 obesity (20), indicating very high body mass relative to stature. Visual assessment confirmed that all participants' high BMI value was due to excessive adipose tissue as opposed to large amounts of muscle mass (see Figure 1 for reference). Research validating kinematics in obese participants is sparse (17, 36) and includes samples with lower average BMI than the current work (34 vs. 42). Therefore, the current findings provide confidence for using skin-based motion capture to study the general population.

### Limitations

There were limitations to this research. First, BMI was used as the primary inclusion criterion, and it is well recognized that BMI does not account for different body compositions. Other measures were not collected to objectively define the composition of lean or adipose tissue of the sample, nor the distribution of soft tissue across areas of interest. Recruitment was difficult for this study, and only nine participants completed both sessions. However, this is only slightly less than the typical sample size for repeatability research (1), indicating these findings are still informative. Accuracy was also not directly assessed in this research, so while differences between calibration procedures are identified, empirical measures to determine which outcomes are most accurate do not exist. However, previous research in these procedures has been conducted (5, 6, 18, 35) and was used to interpret the findings above. Additionally, skin motion artifact of the torso or humeral clusters was not assessed in this study, which could also influence measured scapular kinematics (37). Palpating bony landmarks is a potential source of error in any sample, which may be amplified in this obese sample. To minimize palpation errors, one experienced researcher completed all scapular palpations for double calibration, which is shown to reduce errors (38). Finally, while this approach of assessing orientation at specific humeral elevation levels is common because it allows for scapular kinematic assessment normalized to humeral movement, the specific levels reported in this manuscript (45°, 60°, 75°, 90°) are different than the typical 30° increments (30°, 60°, 90°, 120°) presented (1, 8, 39). This discrepancy was due to the dataset; few datapoints existed at 30° or above 90° for any functional tasks in the current sample. Conversely, previous research with participants with lower BMIs (8, 9, 19) did reach humeral postures of 30° or 120° during some of the functional tasks (i.e., Comb Hair, Forward Transfer). Future research will explore how high BMI influences humeral postures throughout functional tasks.

## Conclusions

Tracking scapular motion with skin-based markers results in acceptable repeatability for upward rotation and internal rotation in people with very high BMIs. Tilt outcomes should be interpreted with caution in these groups. While repeatability was similar between both single and double calibration methods, the double calibration performed marginally better at very high levels of planar elevation. Angle magnitudes were also different between the calibration methods. Further research into the accuracy of scapular motion tracking in participants with high BMI, assessed with objective body composition measures, is required to definitively recommend one calibration method.

## Data Availability

The raw data supporting the conclusions of this article will be made available by the authors, without undue reservation.
